# Supplementary material for: Bystro: rapid online variant annotation and natural-language filtering at whole-genome scale
Source: Genome Biol. 2018 Feb 6;19:14. doi: 10.1186/s13059-018-1387-3 (PMC5801807; doi:10.1186/s13059-018-1387-3)
Supplement: Supplementary file 1 — This file contains: (1) a feature comparison of tested programs; (2) investigation of annotation concordance between tested programs; (3) investigation of Bystro query accuracy. (DOCX 1354 kb) [file 13059_2018_1387_MOESM1_ESM.docx]

Table S1 | **Example of *de novo* variant identification**

| Program | Query (length) | Custom Tools | Variants | Time(s) | | Target Found? |  |
| --- | --- | --- | --- | --- | --- | --- | --- |
| Bystro #1 | 94 characters | 0 | 3 | ~12s | Y | |  |
| Bystro #2 | proband –parents cadd > 20 maf <= .005 |  | 1 | ~12s | **Y** | |  |
| Bystro #3 | proband – parents methemoglobinemia |  | 1 | ~12s | **Y** | |  |
| GEMINI | 154 characters | 1 | 6 | ~540s | Y | |  |
| wANNOVAR | NA | NA | NA | NA | NA | |  |
| VEP | NA | NA | NA | NA | NA | |  |

Bystro was compared to GEMINI (on the Galaxy platform), as well as other recent tools, in filtering a single *de novo* variant in CYB5R3, associated with methemoglobinemia, from a trio dataset provided in GEMINI’s *de novo* search tutorial (<https://s3.amazonaws.com/gemini-tutorials/Gemini-DeNovo-Tutorial.pdf>). Bystro (query #1) and GEMINI queries were written to search for rare variants present only in the proband. Both GEMINI and Bystro were able to find the target variant, but Bystro was able to do so wholly within its search engine, while GEMINI required an additional program (GEMNI’s “de*_*novo” tool). Variants refers to the number of results returned, which equals 1 when only the target variant is found. Bystro returned fewer non-target variants, and was approximately 45x faster. Bystro #2 and #3 demonstrate alternative, shorter queries that return specific results. ANNOVAR/wANNOVAR and VEP were unable to handle *de novo* variant filtering. Bystro query #1 and GEMINI’s query may be found in Supplementary Note 1. ANNOVAR and VEP were unable to handle this task.

**Supplementary Note 1:** Bystro and GEMINI *de novo* queries

The following command was adapted from GEMINI’s *de novo* search tutorial (<https://s3.amazonaws.com/gemini-tutorials/Gemini-DeNovo-Tutorial.pdf> ) and inputted into GEMINI on Galaxy:

gemini de_novo -d 15 --filter *"filter is NULL and is_coding = 1 and impact_severity != ‘LOW’ and (aaf_1kg_eur <= 0.005 or aaf_1kg_eur is NULL) and (aaf_esp_ea <= 0.005 or aaf_esp_ea is NULL)"* trio.trim.vep.denovo.db

The following Bystro query was made within Bystro’s natural-language search engine, after defining “parents” and “proband” synonyms using Bystro’s custom synonyms feature. Bystro’s query used gnomAD as the source of population allele frequency data, instead of Exomes Sequencing Project (ESP) and 1000 Genomes:

*hets:proband -parents ( maf <= .005 || (not in gnomad.genomes not in gnomad.exomes not in dbsnp)) exonic -silent*

Table S2 | **Bystro’s online features compared to other recent programs**

| Feature | Bystro | GEMINI/Galaxy | VEP | wANNOVAR |
| --- | --- | --- | --- | --- |
| **Maximum VCF size** | **> 890GB** | < 66GB | < 763MB | 200MB |
| **Maximum VCF size (.gz)** | **> 129GB** | < 1.3GB | < 13MB | < 8MB |
| **Natural-language search** | **Y** | SQL | Filters | N |
| **Real-time search** | **Y** | N | N | N |
| **Per-query statistics** | **Y** | N | N | N |
| **Search pipelines** | **Y** | N | N | N |
| **Interactive result browser** | **Y** | N | Y | N |
| **Amazon S3 bucket support** | **Y** | Y | N | N |
| **Human & model organisms** | **Y** | hg19 only | Y | hg18 - hg38 |
| **Annotation fields** | **107** | 142 | 65 | 129 (dbNFSP) |
| **Annotates all samples** | **Y** | Y | N | N |
| **Annotates all indel bases** | **Y** | N | N | N |
| **Annotate all transcripts** | **Y** | Y | Y | N |
| **Annotate all variant effects** | **Y** | Y | Y | N |
| **Reports all dbSNP, Clinvar** | **Y** | N | N | N |
| **Genome-wide prediction** | **CADD 1.3** | CADD 1.0 | N | N |
| **Genome-wide conservation** | **Y** | N | N | N |

Bystro offers a modern web application, a powerful search engine, and substantially better performance than existing online variant annotation and filtering applications. In terms of annotation volume, as tested Bystro provides more complete data with fewer output fields. For instance, it provides comprehensive gnomAD and dbSNP 147 annotations, succinctly reporting more complete population allele frequency data than the combination of 1000 Genomes, ExAc, and ESP offered by other programs. wANNOVAR outputs significantly less data than Bystro for most variants, as a plurality of its annotations come from dbNFSP, which has entries for only missense alleles (<1% of the genome). Notably, Bystro also annotates every sample, providing data like zygosity, missingness, and sample minor allele frequency, allowing for variant filtering and QC at per-sample resolution. GEMINI on Galaxy (v0.8.1, Feburary 2016) uses annotation sources that are 1.5 – 3 years outdated, and therefore less complete than Bystro’s. Reported file size maximums are tested values, based on subsets of 1000 Genomes Phase 3. Annotation fields refers to the configuration used in this analysis; VEP can be configured to include additional sources.

Table S3 | **Examples of Bystro handling of ambiguous VCF insertions**

| Variant | POS | REF | ALT |
| --- | --- | --- | --- |
| **Input** | 42680000 | CA | CA |
| Input | 42680000 | CA | CAA |
| Bystro | 42680000 | C\|A | +A |
| Annovar | 42680001-42680001 | - | A |
| VEP | 42680001-42680002 | - | A |

Bystro’s left-shifts VCF alleles to their least-padded representation. Shown is a representative example, variant chr15:42680000CA>CAA. Bystro annotates the CA>CAA allele with 1 base of padding, at the original position 42680000. In comparison ANNOVAR and VEP right-shift the variant by 1 base. The left-shifted position for ANNOVAR would be 42680000-42680000, and for VEP 42680000-42680001, as each program sets the starting position of an insertion as the position preceding the first inserted nucleotide. The full comparisons may be found in Additional files 4, 5, and 6.

Table S4 | **Examples of Bystro handling of complex VCF multiallelic indels**

| Variant | POS | REF | ALT | Genotype |
| --- | --- | --- | --- | --- |
| **Input** | 42680000 | CA | CA |  |
| Input | 107845202 | GACCACC | GACC,G | 0/1 |
| Bystro #1 | 107845203 | A\|C\|C | -3 | het |
| ANNOVAR #1 | 107845206 | ACC | - | het |
| ANNOVAR #2 | 107845203 | ACCACC | - | het |
| VEP #1 | 107845203-107845208 | ACC | - | NA |
| VEP #2 | 107845203-107845208 | ACCACC | - | NA |

Bystro automatically normalizes all alleles at multiallelic sites, and annotates each allele with respect to sample genotypes where provided. Shown is a representative example, for a multiallelic site containing two alleles two alleles, with the 2^nd^ allele missing in all samples (0% frequency). Bystro annotated the parsimonious/left-shifted representation of the first allele, and logged/skipped the 2^nd^, missing variant. ANNOVAR right-shifted the first allele by 3bp, annotated the 2^nd^ allele, and called both alleles as having 50% frequency, despite the 2^nd^ allele being missing. VEP annotated both alleles, and called their positions and lengths as identical. VEP could not output sample information concurrently with –minimal flag, which was used to enable variant normalization. The comparisons may be found in Additional file 4 and Additional file 5.

Figure S1 | **Example of divergent variant annotations**

**
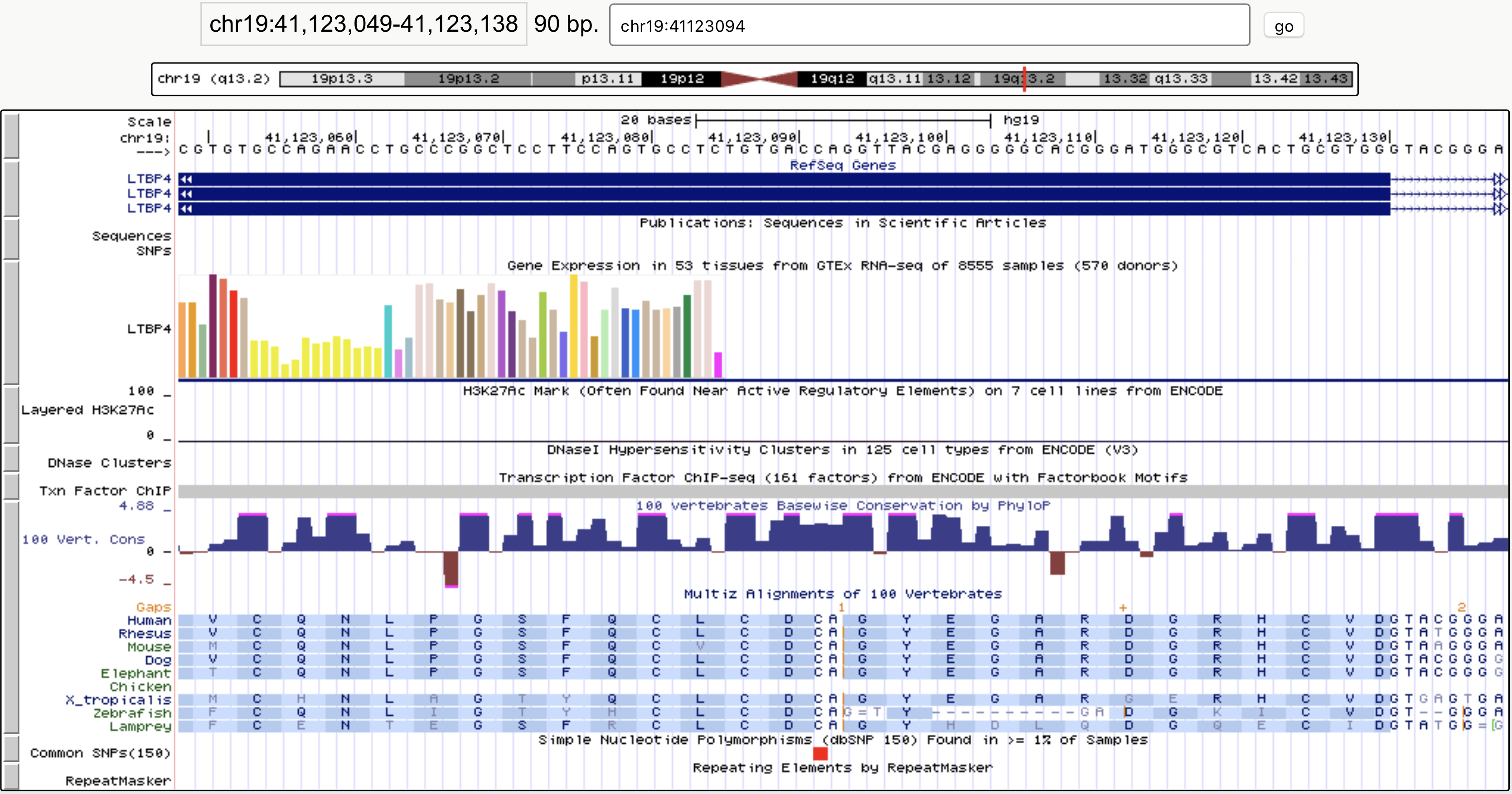
**

| Program | Position Call | Allele Call | Site Type | Consequence |  |
| --- | --- | --- | --- | --- | --- |
| Bystro | 41123094 | +G | exonic;**exonic** | indel-frameshift |  |
| ANNOVAR | 41123094 | G | exonic;**splicing** | Unknown |  |
| SnpEff | NA | NA | NA | frameshift_variant |  |
| VEP | 41123094 | G | NA | frameshift_variant |  |

Bystro annotations were compared to ANNOVAR, VEP, and SnpEff on a small, previously described data set [1]. While most sites had concordant annotations, when results diverged Bystro was correct relative to the underlying transcript data from RefSeq. Shown is a representative example: a 1 base insertion at chr19:41123094G>GG. Bystro, SnpEff, and VEP were completely consistent in their annotations, while ANNOVAR called an erroneous splice site relative to RefSeq, which all programs were configured to use. As seen in both UCSC’s Genome Browser (shown), and the underlying RefSeq transcript data, the nearest intron is 37bp away. SnpEff data was adapted from Yen et al, 2017, where SnpEff’s position and allele calls were not provided. The full comparisons may be found in Additional files 4, 5, and 6.

Table S5 | **Bystro natural-language search specificity on 1000 Genomes data**

| Query | | Variants | % Match Filter | | % Match Custom | |  | |  |
| --- | --- | --- | --- | --- | --- | --- | --- | --- | --- |
| cadd > 15 alt:(A \|\| C \|\| T \|\| G) | 28,099 | |  | 100% | 100% | | |  | |
| gnomad.exomes.af < .001 cadd > 15 missense | 6,840 | |  | 100% | 100% | | |  | |
| gnomad.exomes.af < .001 cadd > 15 nonsynonymous | 6,840 | |  | 100% | 100% | | |  | |
|  | |  |  |  | |  | |  |  |
| cadd > 15 | | 29.057 |  | 100% | | 100% | |  |  |
| alt:(A \|\| C \|\| T \|\| G) | | 963,802 |  | 100% | | 100% | |  |  |
| gnomad.exomes.af < .001 | | 30,674 |  | 100% | | 100% | |  |  |
| missense | | 16,326 |  | 100% | | 100% | |  |  |
| nonsynonymous | | 16,326 |  | 100% | | 100% | |  |  |

The accuracy of Bystro natural-language queries were assessed on 1 million variants from 1000 Genomes Phase 3 chromosome 1. “% Match Filter” refers to the overlap between the variants returned by Bystro’s “Filters”, which are an exact-match search feature, and the natural-language search queries listed. “% Match Custom” refers to the overlap between the variants returned by custom Perl filtering scripts and the natural language search queries listed. In all cases the natural-language queries matched exactly, both by number of variants returned, as well as their identity, as judged by a Linux “diff” command. The individual components of each query were also tested and found to be specific. We note that it is more likely for very short queries, such as a single word that is often present in multiple annotation fields, to produce unexpected matches. This is addressable by specifying the field to search within (i.e “refSeq.siteType:intergenic” instead of “intergenic”), or by using Bystro “Filters”. All scripts created and used may be found at <https://github.com/akotlar/bystro-paper>.

Table S6 | **Bystro natural-language search specificity compared with Excel filters**

| Query | | Variants | % Match Filter | | % Match Excel |
| --- | --- | --- | --- | --- | --- |
| cadd > 15 alt:(A \|\| C \|\| T \|\| G) | 43 | |  | 100% | 100% |
| gnomad.exomes.af < .001 cadd > 15 missense | 6 | |  | 100% | 100% |
| gnomad.exomes.af < .001 cadd > 15 nonsynonymous | 6 | |  | 100% | 100% |

Bystro natural-language queries were compared for concordance with Bystro’s exact-match “Filters”, as well as Excel filters, on a previously described dataset [1]. “% Match” refers to the degree of overlap between the natural-language search query and the exact-match filtering method. The full comparison may be found in Additional file 7.

**References**

1. Yen JL, Garcia S, Montana A, Harris J, Chervitz S, Morra M, West J, Chen R, Church DM: **A variant by any name: quantifying annotation discordance across tools and clinical databases.** *Genome Med* 2017, **9:**7.
